# Quantitative dynamic contrast-enhanced magnetic resonance imaging in head and neck cancer: A systematic comparison of different modelling approaches

**DOI:** 10.1016/j.phro.2024.100548

**Published:** 2024-02-09

**Authors:** Marte Kåstad Høiskar, Oddbjørn Sæther, Mirjam Delange Alsaker, Kathrine Røe Redalen, René M. Winter

**Affiliations:** a Department of Physics, Norwegian University of Science and Technology, Trondheim, Norway; b Department of Radiology and Nuclear Medicine, St. Olavs Hospital, Trondheim University Hospital, Trondheim, Norway; c Cancer Clinic, St. Olavs Hospital, Trondheim University Hospital, Trondheim, Norway

**Keywords:** Dynamic contrast-enhanced MRI, Arterial input function, Pharmacokinetic modelling, T staging, Head and neck cancer

## Abstract

**Background and purpose:**

Dynamic contrast-enhanced magnetic resonance imaging (DCE-MRI) describes tissue microvasculature and has prognostic and predictive potential in radiotherapy for head and neck cancer (HNC). However, lack in standardization of DCE-MRI hinders comparison of studies and clinical implementation. This study investigated the accuracy and robustness of the population arterial input function (AIF), correlations between pharmacokinetic parameters and their association to T stage and human papillomavirus (HPV) status for HNC.

**Materials and methods:**

DCE-MRI was acquired for 44 HNC patients. Population AIFs were calculated with six different approaches. DCE-MRI was analysed in primary and lymph node tumours using Tofts model (TM) with population AIFs and individual AIFs, extended TM (ETM) with individual AIFs, Brix model (BM), and areas under the curve (AUCs). Intraclass correlation, concordance correlation, Pearson correlation and Whitney Mann *U* test helped examining the robustness and accuracy of population AIF, correlations between DCE-MRI parameters and their association to T stage and HPV status, respectively.

**Results:**

The population AIF was robust but differed from individual AIFs. There was significant correlation between K^trans^_TM/ETM_ and v_e, TM/ETM_, and K^trans^_TM/ETM_ and K_ep, TM/ETM_. A_Brix_ and AUCs correlated for lymph nodes. K_ep, Brix_ correlated with A_Brix_, K^trans^_TM/ETM_ and K_ep, TM/ETM_ for primary tumours. K_ep, TM_ significantly decreased with increasing T stage. Both the correlations and the parameters’ association to T stage were stronger for HPV negative lesions.

**Conclusions:**

Individual AIF was preferred for accurate pharmacokinetic modelling of DCE-MRI. DCE-MRI parameters and their correlations were affected by the lesion type, HPV status and T staging.

## Highlights

• Population arterial input function (AIF) was robust, but not accurate.
• Population AIF did not vary with different data pre-processing methods.
• Individual AIF was preferred over population AIF for accurate Tofts modelling.
• Pharmacokinetic parameters varied with human papillomavirus status and lesion type.
• K_ep_ from the Tofts model significantly decreased with increasing T stage.

## Graphical abstract

**Figure:**
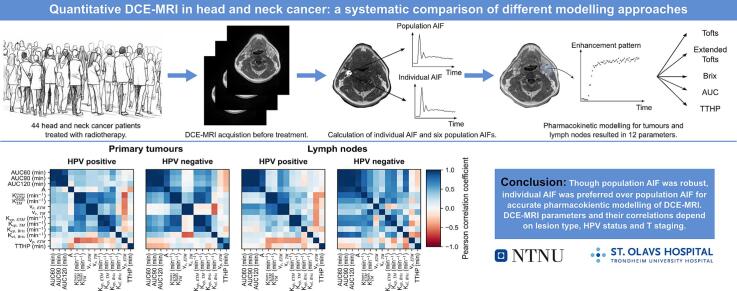

## 1 Introduction

Head and neck cancer (HNC) is the sixth most common cancer worldwide, with half of the patients undergoing radiotherapy [1], [2]. Despite advances in treatment techniques, the 5-year survival rate of HNC has remained around 60 % [3], and many patients still experience side-effects [4]. To improve treatment outcome and patient survival, current research focuses on individualising radiotherapy by utilising quantitative imaging techniques.

Dynamic contrast-enhanced magnetic resonance imaging (DCE-MRI) is a promising quantitative imaging modality for assessing tissue response to radiation [5], [6]. DCE-MRI involves image acquisitions before, during and after intravenous injection of a contrast agent (CA). The measured time-intensity curves (TICs) can be analysed with pharmacokinetic models, resulting in parameters reflecting information on tissue microvasculature and microenvironment, such as perfusion, permeability, and hypoxia [7], [8]. Tissue microvasculature and hypoxia can influence tissue response to radiation [9], and may serve as prognostic or predictive biomarkers. Parameter maps from voxel-wise calculations may capture tumour heterogeneity better and allow definition of intra-tumoural targets for dose painting [6]. However, most studies have reported associations between mean parameter distributions and radiotherapy outcome [10]. If an unfortunate outcome is predicted, the course of treatment can be changed to improve it.

The lack of standardisation of quantitative DCE-MRI analysis poses a challenge to clinical implementation [11], study comparisons and evaluation of pharmacokinetic parameters. Some models, e.g. the Tofts model (TM), require an arterial input function (AIF) to calculate pharmacokinetic parameters [12]. Considerable research effort has been devoted to developing accurate methods for AIF quantification. Besides direct blood sampling or reference tissue approaches, a common method is image-derived AIF quantification from an imaged arterial blood vessel near the tumour [11], [13], [14]. Accurate and robust AIF estimation is important for reliable parameter estimation. While individual AIF based on the arterial TIC of the individual patient is commonly used, a population AIF based on the arterial TICs of multiple patients may be needed if individual AIF is hindered. The parameters derived with a population AIF may have higher repeatability, as found in abdominal cancer [15]. Additionally, some HNC studies have shown that parameters calculated with population AIF were close to the ones calculated with individual AIFs, though the patient cohort was small [16], [17]. However, variations in calculation of image-derived population AIFs can also result in significantly different pharmacokinetic parameters [18], [19]. Therefore, more robustness analyses of population AIF estimation are needed to reach a standardised method [20].

DCE-MRI analysed with different pharmacokinetic models [12], [21], [22] hinders comparisons. While studies have examined correlations between parameters within one model [23], no studies have compared different models for HNC. A better understanding of the relations between models could help compare and interpret results. The large number of models also results in many candidates for prognostic or predictive biomarkers. The most promising DCE-MRI biomarker for HNC is K^trans^ from TM, describing the transfer of contrast from the plasma space to the extracellular extravascular space (EES), but there are potentially additional useful biomarkers [6]. A more thorough comparison of DCE-MRI parameters from different models could help select which parameters merit further investigation.

This study aimed to investigate the accuracy and robustness of a population AIF in HNC, and the relation between pharmacokinetic and semi-quantitative parameters from different models and their association to relevant clinical factors.

## 2 Materials and methods

### 2.1 Patients

In this study, 44 head and neck squamous cell carcinoma patients were included. MRI was acquired before radiotherapy, sometimes together with chemotherapy, at St. Olavs Hospital in Trondheim, Norway. The patient and tumour characteristics are described in Table 1. The study was approved by the Regional Committee for Medical Research Ethics in Central Norway (approval number 2019/64744) and all patients gave their written informed consent.

**Table 1:** Patient and tumour characteristics. HPV; human papilloma virus.

**Age (years)**	
Range	53–86
Median	68
**Sex assigned at birth**	
Female	31
Male	13
**Lesions**	
Primary tumours	36
Lymph nodes	44
**T stage**	
T1	5
T2	14
T3	9
T4	7
**HPV status (no. of lesions)**	
HPV positive (primary tumours/lymph nodes)	20/32
HPV negative (primary tumours/lymph nodes)	16/12

### 2.2 MRI acquisition

MRI was performed on a 1.5 T MR scanner (Magnetom Avanto Fit, Siemens Healthineers, Erlangen, Germany) with a 20-channel head and neck coil. A T1-weighted spoiled gradient-echo (SPGR) sequence was acquired with five flip angles to calculate maps of longitudinal relaxation time T1 prior to contrast injection (T10 maps). Radiofrequency transmit field (B1) maps were measured with a proton weighted SPGR sequence and were used for T1 map corrections.

For the DCE-MRI, the patients were injected intravenously with 0.2 ml/kg body weight of the gadolinium-based CA gadoterate meglumine (Clariscan) at a rate of 3 ml/s followed by a 20 ml saline flush. The CA was given automatically three timeframes after the DCE-MRI acquisition started. The DCE-MRI sequence was a T1-weighted SPGR sequence and consisted of 60 consecutively acquired axial volumes (0.78x0.78x4 cm^3^) with a temporal resolution of 3.735 s. The scanning parameters for T1 map, B1 map and DCE-MRI are listed in Supplementary Table S1.

### 2.3 Arterial input function

The TM and extended TM (ETM) require an AIF to calculate pharmacokinetic parameters. An individual AIF was derived for each patient by manually delineating the left carotid artery at the time of maximum arterial signal intensity. The 5 % most enhanced voxels in the delineated artery were selected as arterial region of interest (ROI) to calculate the AIF, as recommended by the Quantitative Imaging Biomarkers Alliance [24]. The signal in the arterial ROI was averaged and converted to contrast concentration [12]:

(1) $$C\left(t\right)=\frac{1}{r_{1}}\left(\frac{1-k\frac{S\left(t\right)}{S_{0}}}{1-k\cos\alpha \frac{S\left(t\right)}{S_{0}}}\right)\;where\;k=\frac{1-e^{-TR/T_{10}}}{1-\cos\alpha e^{-TR/T_{10}}},$$

resulting in a time-concentration curve (TCC) called individual AIF. Here, S(t) is the TIC, r_1_ = 3.1 ${\left(smM\right)}^{-1}$ is the specific relaxivity, α is the flip angle, TR is the repetition time, T_10_ is the measured T10 map and S_0_ is the average signal before CA injection.

**Fig. 1:**
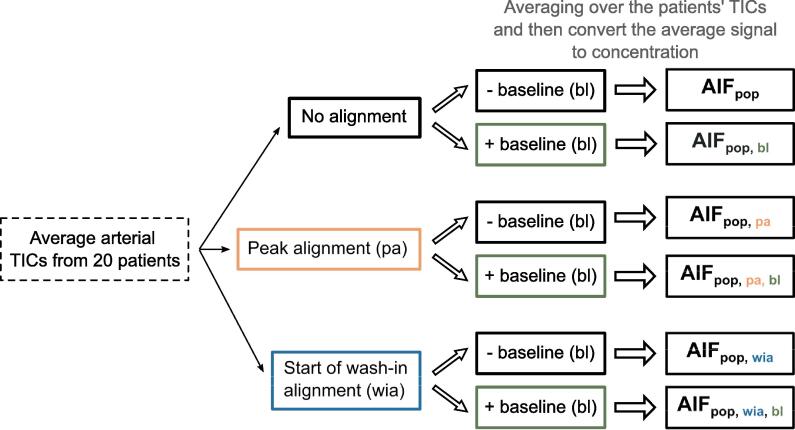
Flowchart of how the six different population arterial input functions (AIFs) were calculated. The arterial time-intensity curves (TICs) from 20 patients were obtained. The arterial TICs were either not aligned, aligned by their peaks (pa), or aligned by the start of wash-in (wia). The baseline (bl), i.e. the four time frames before bolus injection, of the arterial TICs were either removed or kept for each alignment method. For each combination of alignment method and baseline removal/inclusion, the patients’ arterial TICs were averaged over, and the signal was converted to concentration, resulting in six different population AIFs.

Population AIFs were measured with six different methods as illustrated by Fig. 1. The arterial TICs from 20 patients were either not aligned, aligned by their peaks, or aligned by the start of wash-in, i.e., start of increase in signal intensity. For each alignment method, the signal before CA injection, called the baseline of the TICs, was either removed or kept. The patient arterial TICs were then averaged, resulting in a single population TIC for each alignment and baseline approach. The population TICs were converted to TCCs by Equation (1), generating six population AIFs in their measured form: AIF_pop_, AIF_pop, bl_, AIF_pop, pa_, AIF_pop, pa, bl_, AIF_pop, wia_, AIF_pop, wia, bl_. The subscripts pop, pa, wia, bl stand for population, peak alignment, wash-in alignment, and baseline included, respectively. Parker’s equation [15] was fitted to each of the population AIFs to find their functional form (Supplementary Material, Section 2).

### 2.4 Pharmacokinetic modelling

The lesions were manually delineated in the 20th timeframe of the DCE-MR images with 3DSlicer (slicer.org). Delineations were validated or manually adjusted by a radiation oncologist. Quantitative and semi-quantitative DCE-MRI analysis was performed for each lesion with an in-house developed Python script (https://github.com/martekhoiskar/qDCE_MRI).

Pharmacokinetic parameters were calculated using the TM, ETM and Brix model (BM) for all lesions. TM, ETM and BM are two-compartment models that describe the transfer of CA between the plasma space and EES.

According to TM, the concentration of CA in the tissue at time t is given by:

(3) $$C_{t}\left(t\right)=K^{\mathrm{trans}}∙\int_{0}^{t}C_{a}\left(\tau \right)exp\left(-\frac{K^{\mathrm{trans}}}{v_{e}}\left(t-\tau \right)\right)d\tau$$

where C_a_(t) is an AIF in measured or functional form, K^trans^ is the volume transfer constant, and v_e_ is the EES volume fraction [12]. While TM assumes the plasma volume fraction is negligible, ETM considers the plasma volume fraction, v_p_, in an additional term [25]:

(4) $$C_{t}\left(t\right)=K^{\mathrm{trans}}∙\int_{0}^{t}C_{a}\left(\tau \right)exp\left(-\frac{K^{\mathrm{trans}}}{v_{e}}\left(t-\tau \right)\right)d\tau +v_{p}C_{a}\left(t\right).$$

BM does not require an AIF and describes the time course of signal intensity S(t) by:

(5) $$\frac{S\left(t\right)}{S_{0}}=1+\frac{A}{k_{\mathrm{ep}}-k_{\mathrm{el}}}∙\left(\frac{\left(exp\left(k_{\mathrm{el}}t^{\prime }\right)-1\right)∙exp\left(-k_{\mathrm{el}}t\right)}{k_{\mathrm{el}}}-\frac{\left(exp\left(k_{\mathrm{ep}}t^{\prime }\right)-1\right)∙exp\left(-k_{\mathrm{ep}}t\right)}{k_{\mathrm{ep}}}\right)$$

where t’ = t before and during contrast injection (t < τ) and t’ = τ after contrast injection (t > τ). Here, S_0_ is the average signal intensity before contrast injection, A is a scalar constant, k_ep_ is the transfer rate of CA from the EES to plasma space, and k_el_ is the clearance rate of CA from the plasma space [21].

To calculate TM parameters based on mean tumour TIC, for each lesion, the mean TIC of the voxels was converted to TCC by Equation (1). The TCC was repeatedly fitted to Equation (3) using the measured individual AIF and all six population AIFs in their functional form, resulting in seven sets of TM parameters per lesion. The TCC was fitted to Equation (4) using the measured individual AIF to obtain the ETM parameters. The rate constant, K_ep_, was calculated using the relation K_ep_ = K^trans^/v_e_. BM parameters were calculated by fitting the mean TIC to Equation (5).

### 2.5 Semi-quantitative calculations

Areas under the curve (AUCs) and the time-to-half-peak (TTHP) were calculated for each lesion. AUC60, AUC90 and AUC120 were defined as areas under the contrast index (CI) curve for the first 60, 90 and 120 s after CA arrived in the lesion [26]. The CI curve was obtained:

(6) $$CI\left(t\right)=\frac{S\left(t\right)-S_{0}}{S_{0}}.$$

AUC60, AUC90 and AUC120 were calculated by integrating CI(t) using Simpson’s rule.

TTHP was defined as the time from the CA injection to signal intensity reached half its maximum value [27]. After finding the half maximum, S_max_ /2, of the mean TIC, the two closest time points were used to obtain the intercept and slope of the connecting line. TTHP was calculated using the relation:

(7) $$\mathrm{TTHP}=\frac{S_{\max}/2-Intercept}{\mathrm{Slope}}$$

The pharmacokinetic and semi-quantitative analysis was also performed voxel-wise and is described in Supplementary Material Section 4.

### 2.6 Statistical analysis

The agreement between the mean TM parameters calculated with different population AIFs was quantified by the intraclass correlation coefficient (ICC). The two-way mixed-effects model for single measures with absolute agreement was used [28]. The concordance correlation coefficient (CCC) was calculated to assess the agreement between TM parameters found using AIF_pop_ against the ones found with individual AIFs. All lesions were included.

The Pearson correlation coefficients (PCCs) between different pairs of mean pharmacokinetic (TM, ETM, BM parameters) and semi-quantitative (AUC60, AUC90, AUC120, TTHP) parameters were calculated, together with their corresponding p-values. Here and in the following analysis, TM and ETM parameters were those obtained with the individual AIF. The PCC analysis was performed for all lesions, primary tumours only, and lymph nodes only. All statistical analysis was performed in Python (v3.11, packages SciPy 1.10.1, pyirr 0.84.1.2).

The parameters’ association to T stage was investigated with a Mann-Whitney *U* test, hypothesising a difference in parameter distribution between different T-stage stratified groups. The test was performed using SPSS (v29.0.0.0) with 5 % significance level. Both the PCC and Mann-Whitney U analysis was performed for groups of human papillomavirus (HPV) positive and negative lesions together, and separately.

## 3 Results

**Fig. 2:**
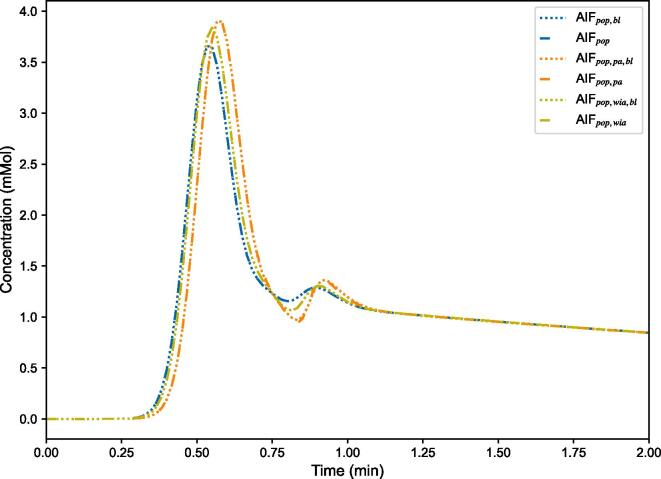
The six different population arterial input functions (AIFs) plotted against time. The population AIFs with the subscript bl had their baseline included while calculating the parameter values. The subscript pa and wia means the individual AIFs were aligned by their peaks and start of enhancement, respectively, before averaging over the patients’ individual AIFs to find the population AIF.

Population AIFs are plotted in their functional form in Fig. 2. The mean and relative standard deviation of the population AIF parameters describing their functional forms are listed in Supplementary Table S2.

The ICCs of K^trans^_TM_ and v_e, TM_, obtained with the different population AIFs, were 0.83 and 0.98, respectively. The CCCs of K^trans^_TM_ and v_e, TM_ obtained with AIF_pop_ and individual AIF were 0.46 and 0.45, respectively.

**Fig. 3:**
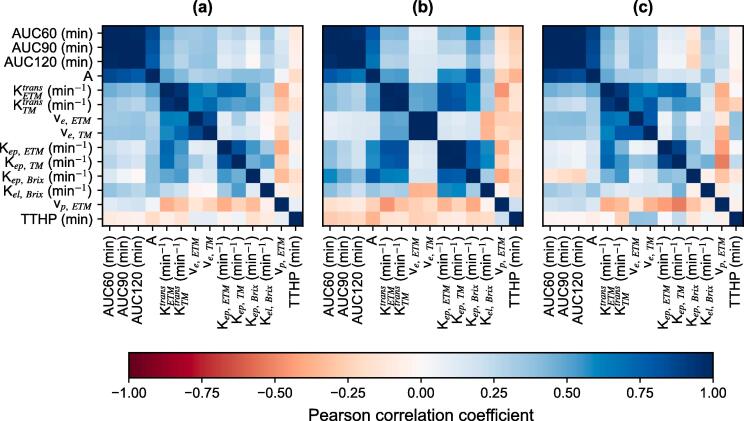
Heatmaps showing the Pearson correlation coefficients between pairs of different pharmacokinetic and semi-quantitative parameters for a) all lesion, b) primary tumours and c) lymph nodes.

The PCC is visualised by heat maps in Fig. 3, for all lesions (a), primary tumours only (b) or lymph nodes only (c). The PCC between K^trans^, v_e_ and K_ep_ from TM and their corresponding, eponymous parameter from ETM were 0.94, 0.86 and 0.83 for all lesions, 0.98, 1.00 and 0.99 for primary tumours, and 0.92, 0.79 and 0.88 for lymph nodes, respectively.

Additional correlations > 0.75 were found for primary tumours or lymph nodes only. For primary tumours, K_ep, Brix_ correlated with A_Brix_ and with K_ep_ from both TM and ETM with a PCC of 0.78, 0.77 and 0.78, respectively. Additionally, K^trans^ correlated with K_ep_ within both TM and ETM with a PCC of 0.81 and 0.82, respectively. For lymph nodes, A_Brix_ correlated with all AUC metrics with a PCC ≥ 0.84, and K^trans^_TM_ correlated with v_e, TM_ with a PCC of 0.79.

**Fig. 4:**
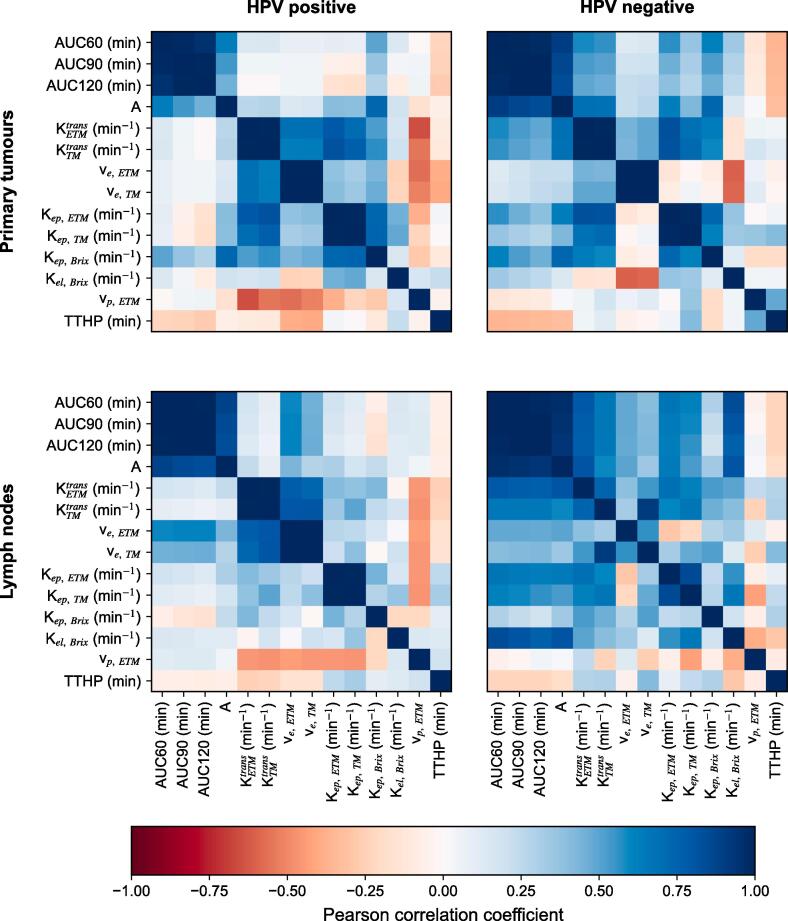
Heatmaps showing the Pearson correlation coefficient between pairs of different pharmacokinetic and semi-quantitative parameters for human papilloma virus (HPV) positive primary tumours (upper left), HPV negative primary tumours (upper right), HPV positive lymph nodes (lower left) and HPV negative lymph nodes (lower right).

The correlation between the parameters was different when examining HPV positive and negative lesions separately. Despite a few exceptions, Fig. 4 shows that the PCCs were generally stronger for HPV negative lesions compared to positive ones, both for primary tumours and lymph nodes.

Some correlations within primary or lymph node tumours were not revealed until the HPV status stratification. This included correlations between A_Brix_ and the AUC metrics which emerged for HPV negative primary tumours (PCCs ≥ 0.84), as well as correlations between K^trans^_ETM_ and the AUCs and A_Brix_, and between K_el, Brix_ and the AUCs and A_Brix_, which emerged for HPV negative lymph nodes (PCCs ≥ 0.76). All PCCs listed above were statistically significant (Supplementary Fig. S1 and S2).

**Fig. 5:**
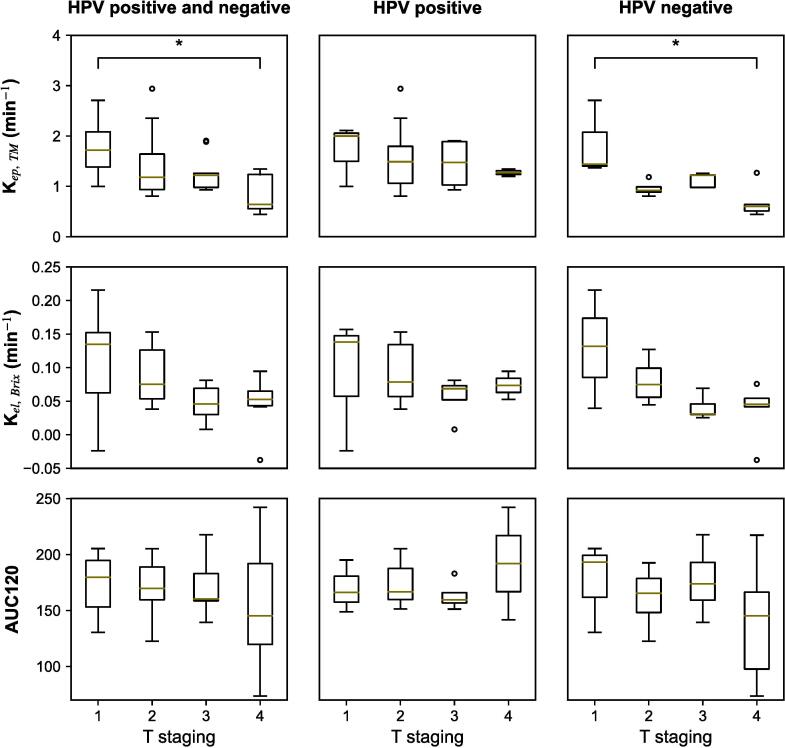
Box plot showing the difference between K_ep, TM_ (first row), K_el, Brix_ (second row) and AUC120 (third low) in primary tumours among T staging for both human papillomavirus (HPV) positive and negative lesions (left column), only HPV positive lesions (middle column) and only HPV negative lesions (right column). The star sign indicating statistically significant results for the Mann-Whitney *U* test at the 5 % level. AUC120; area under the curve up to 120 s after contrast injection.

Fig. 5 illustrates the associations between T stage of primary tumours and K_ep, TM_, K_el, Brix_ and AUC120 when including both HPV positive and negative, only HPV positive or only HPV negative tumours. The figure shows a trend of decreasing parameter values with increasing T stage. The largest difference was seen between T1 tumours and tumours of T stage ≥ 2, but the difference was not statistically significant for any parameter in Fig. 5, except for K_ep, TM_ between T1 and T4 tumours when both HPV positive and negative tumours (p = 0.008) and when only HPV negative tumours (p = 0.036) were included. Though the differences between T stages were not significant for most parameters, they were larger for HPV negative tumours compared to positive ones.

## 4 Discussion

We analysed DCE-MRI of HNC patients using three pharmacokinetic models with six different population AIFs and individual AIFs. Though different data pre-processing approaches did not affect the population AIF and resulting TM parameters, individual AIF was preferred for accurate pharmacokinetic parameter calculation. Some parameters from different models correlated with each other, but it varied with lesion type and HPV status. The HPV status also affected the parameters’ association to T staging.

Since the variability of K^trans^_TM_ and v_e, TM_ obtained with different population AIFs was low (ICCs ≥ 0.83), the population AIF and resulting TM parameters were not affected by alignment method and removal of baseline. Other AIF calculation steps, such as artery segmentation method [29], [30] and AIF scaling [31], are more important and need to be standardised. Contrary to our results, Onxley et al. [16] and Shuckla-Dave et al. [17] found that TM parameters calculated with population and individual AIF did not differ for HNC, but their results were from small patient cohorts. Although much remains before DCE-MRI can be standardised, the difference in pharmacokinetic parameters due to AIF variations are often systematic, resulting in similar parametric maps that still depict tumour heterogeneity and changes in parameters over time [30], [32].

According to Andersen et al. [33], A_Brix_ correlated with K_el, Brix_, K^trans^_TM_ and v_e, TM_, and K_el, Brix_ with v_e, TM_ for locally advanced cervical cancer, while we only saw correlation between A_Brix_ and K_el, Brix_ for HPV negative lymph nodes, between A_Brix_ and K^trans^_TM_, and between K_el, Brix_ and v_e, TM_ for HPV negative primary tumours for HNC. High heterogeneity between cancer types, could explain these partly divergent findings. In addition, Andersen et al. [33] used a reduced expression of BM for bolus injection and parameter constraints during curve-fitting, and TM parameters were calculated with population AIF, which could explain the differences to our results.

Contrary to our results, Roberts et al. [34] and Chih-Feng et al. [35] found that AUC60 correlated with K^trans^_TM_ and v_e, TM_ for brain tumours. On the other hand, Roberts et al. [34] found that AUC60 correlated only with v_e, TM_ for abdominal tumours, suggesting that correlations differ between cancer types. A simulation study has also shown that AUC only correlates with K^trans^_ETM_, v_e, ETM_ and v_p, ETM_ under certain conditions [36]. As an example, AUC is proportional to K^trans^_ETM_ when K^trans^_ETM_ is small and v_e, ETM_ is large. The conditions were not found in our study and may explain the lack of such correlations. Although AUCs can easily be calculated, they have no clear physiological meaning [37]. Therefore, quantitative parameters may be more useful.

Studies have shown that K_ep, TM_ and K_el, Brix_ correlate positively with tumour microvessel density (MVD) [38], [39], and that K_ep, TM_ correlates negatively with tumour hypoxia [40]. Aggressive tumours are often associated with increased hypoxia and reduced MVD [41], [42], which can explain why K_ep, TM_ and K_el, Brix_ decreased with increasing T stage. Our results regarding K_ep, TM_ agreed with Guo et al. [43] but differed from Leifels et al. [44]. Although Andersen et al. [33] found that K_el, Brix_ is a potential prognostic biomarker for locoregional control for cervical cancer, no other studies, to our knowledge, have reported the correlation between K_el, Brix_ and T stage for HNC. While several studies have found that K^trans^_TM_ is a promising prognostic and predictive biomarker for HNC [33], [45], [46], [47], K^trans^_TM_ did not correlate with T stage in our study. However, K^trans^_TM_ showed a weak negative trend with increasing T stage, especially for HPV negative tumours. The true clinical value of K_ep, TM_, K_el, Brix_ and K^trans^_TM_ will be further investigated when long-term outcome is available.

There are limitations to this study. It is not possible to conclude which model produces the most valid parameter values. Although phantoms cannot perfectly emulate true tissue characteristics, DCE-MRI flow phantom studies, such as conducted by Foltz et al. [48], may yield insights into which AIF and model that best describe the microvasculature. Multicentre DCE-MRI phantom studies, like the study by van Houdt et al. [49], are recommended to investigate the robustness of the models across institutions [14]. Both Foltz et al. [48] and Houdt et al. [49] found that phase data can measure AIF and CA concentration more accurately than magnitude data. Another limitation was low statistical power. We examined possible parameter associations to T stage for HPV negative and positive lesions, separately, but this resulted in smaller groups and low statistical power. Therefore, larger cohorts are required to confirm our findings.

To conclude, the population AIF was not affected by pre-processing methods, but the individual AIF was still preferred for accurate pharmacokinetic parameter calculation. The parameters and correlations between them were affected by lesion type, HPV status and T staging. Follow-up data of HNC patients will later be used to investigate the prognostic potential of K_ep, TM_, K_el, Brix_ and K^trans^_TM._

## CRediT authorship contribution statement

**Marte Kåstad Høiskar:** Conceptualization, Formal analysis, Investigation, Methodology, Visualization, Writing – original draft, Writing – review & editing. **Oddbjørn Sæther:** Methodology, Resources, Writing – review & editing. **Mirjam Delange Alsaker:** Investigation, Formal analysis, Writing – review & editing. **Kathrine Røe Redalen:** Conceptualization, Funding acquisition, Methodology, Supervision, Writing – original draft, Writing – review & editing. **René M. Winter:** Methodology, Supervision, Writing – original draft, Writing – review & editing.

## Declaration of competing interest

The authors declare that they have no known competing financial interests or personal relationships that could have appeared to influence the work reported in this paper.
