# Supplementary material for: Quantitative dynamic contrast-enhanced magnetic resonance imaging in head and neck cancer: A systematic comparison of different modelling approaches
Source: Phys Imaging Radiat Oncol. 2024 Feb 9;29:100548. doi: 10.1016/j.phro.2024.100548 (PMC10876686; doi:10.1016/j.phro.2024.100548)
Supplement: Supplementary data 1 [file mmc1.docx]

Supplementary Material

# MRI acquisition

The map of the radiofrequency transmit field (B1 map), the map of the longitudinal relaxation time T1 prior to contrast injection (T10 map), and dynamic contrast-enhanced magnetic resonance imaging (DCE-MRI) was acquired. The scanning parameters of the B1 map, T10 map and DCE-MRI are listed in Table S1.

Supplementary Table S1: Magnetic resonance imaging (MRI) parameters for acquiring the T10 map using variable flip angle method, the B1 map and the dynamic contrast-enhanced MRI (DCE-MRI). T10 map: map of longitudinal relaxation time T1 prior to contrast injection, B1 map (radiofrequency transmit field), TR; repetition time, TE; echo time.

|  | **T10 map** | **B1 map** | **DCE-MRI** |
| --- | --- | --- | --- |
| **TR (ms)** | 4.76 | 3800 | 3.04 |
| **TE (ms)** | 1.95 | 1.88 | 1.11 |
| **Flip angle (degrees)** | 2, 10, 15, 20, 25 | 79.99 | 12 |
| **Matrix size** | 154x192 | 52x64 | 288x320 |
| **Slices** | 48 | 16 | 22 |
| **Voxel size (mm^3^)** | 11.68x1.35x4 | 5.92x5.94x8 | 0.78x0.78x4 |
| **Averages** | 1 | 1 | 1 |

# Population AIF

In the study, six different population arterial input functions (AIFs) were calculated using different pre-processing approaches.

Parker et al. [14] suggested a parameterization of the population AIF to obtain a functional form:

|  | $C_{a}(t) = \sum\frac{A_{n}}{\sigma_{n}\sqrt{2\pi}}exp\left( -\left( t-T_{n} \right)^{2}/2\sigma_{n}^{2} \right) + \alpha\frac{exp(-\beta t)}{1+exp\left( -s\left( t-\tau\right) \right)}$ | (1) |
| --- | --- | --- |

where A_n_, T_n_ and $\sigma_{n}$ are the scaling constants, centres, and widths of an nth Gaussian, $\alpha$ and $\beta$ are the amplitude and decay constant of an exponential, and $\tau$ and s are the centre and width of a sigmoid function. Equation (1) was fitted to each of the six population AIFs using non-linear least squares to find their functional form.

Hence, the function form of the populations AIFs was described by 10 parameters: A_1_, A_2_, T_1_, T_2_, $\sigma$_1_, $\sigma$_2_, $\alpha$, $\beta$, s and $\tau$. The results of the mean and relative standard deviation of the population AIF parameters describing their functional form are listed in Table S2.

Table S2: The population AIFs are described by ten parameters suggested by Parker et al. [14] and the mean of the parameters and their corresponding relative standard deviations are listed. SD: standard deviation.

| **Parameter** | **Mean value** | **SD (%)** |
| --- | --- | --- |
| A_1_ (mMol.min) | 0.55 | 1.8 |
| A_2_ (mMol.min) | 0.382 | 2.4 |
| T_1_ (min) | 0.55 | 1.8 |
| T_2_ (min) | 0.71 | 1.4 |
| $\sigma_{1}$ (min) | 0.0664 | 0.7 |
| $\sigma_{2}$ (min) | 0.125 | 1.6 |
| $\alpha$ (mMol) | 1.374 | 0.2 |
| $\beta$ (min^-1^) | 0.2429 | 0.4 |
| s (min^-1^) | 39 | 20.5 |
| $\tau$ (min) | 0.86 | 1.2 |

# Pearson correlation between DCE-MRI parameters

The Person correlation coefficient (CC) between pairs of pharmacokinetic and semi-quantitative parameters were calculated for all lesions, primary tumours only and lymph nodes only. The corresponding p-values are visualised in Figure S1. In addition, the Pearson CC were calculated for HPV positive and negative lesions, separately, for both primary tumours and lymph nodes. Figure S2 show the corresponding p-values to the Pearson CCs.


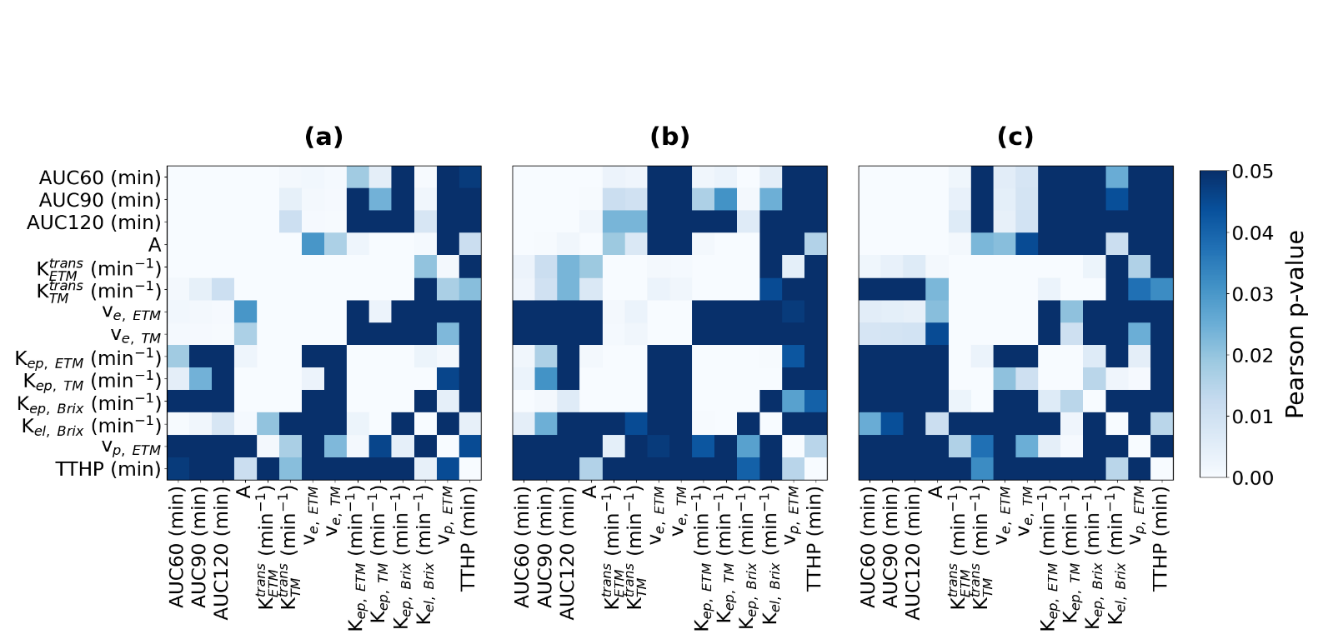


Supplementary Figure S1: Heatmaps showing the p-values corresponding to the Pearson correlation coefficients between both the pharmacokinetic parameters and the semi-quantitative parameters for a) all lesion, b) primary tumours and c) lymph nodes.


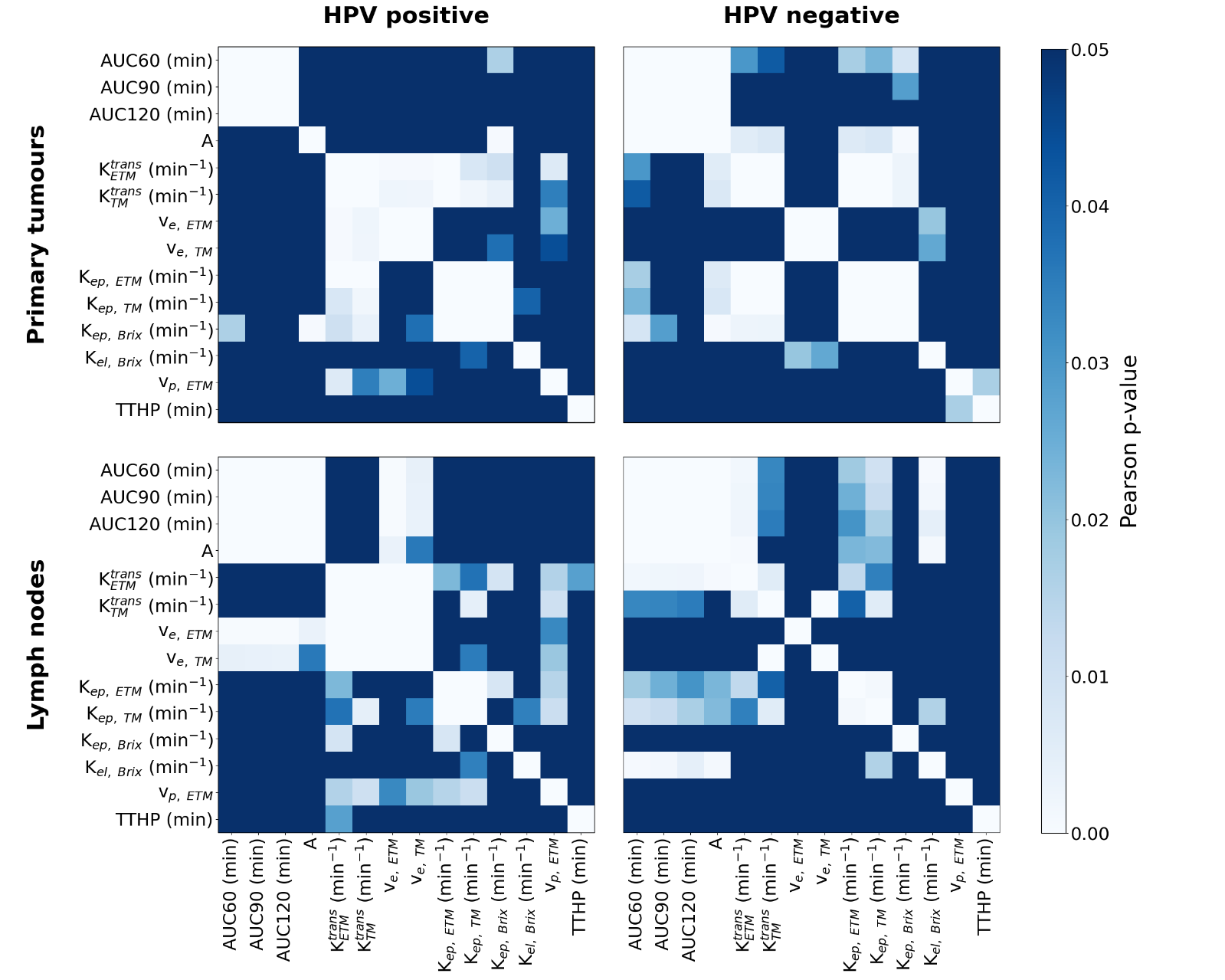


Supplementary Figure S2: Heatmaps showing the p-values corresponding to the Pearson correlation coefficients between both the pharmacokinetic parameters and the semi-quantitative parameters for human papillomavirus (HPV) positive primary tumours (upper left), HPV negative primary tumours (upper right), HPV positive lymph nodes.

# 4. Voxel parameter distributions

In addition to the lesion mean, the pharmacokinetic and semi-quantitative analysis were also performed voxel-wise, resulting in 14 parameter maps per lesion. To analyse intra-tumour heterogeneity, the voxel parameter distributions within the lesion were analysed using standard deviation (std) and skewness. To analyze differences between different tumour groups, the distribution of these metrics was compared with Mann-Whitney U test between groups of all lesions, stratified by lesion type (primary tumour, lymph node), stratified by HPV status (negative, positive), and stratified by both lesion type and HPV status (negative primary tumours, positive primary tumours, negative lymph nodes, positive lymph nodes). In addition, changes of std and skewness with T stage was analysed with Mann-Whitney U test for groups of all lesions, HPV positive, and HPV negative lesions.

An example of the histogram of one parameter, K_ep, TM,_ in primary tumour and malignant lymph node of a patient is given in Figure S3. The distributional differences in std and skewness of K_ep, TM_ between the different tumour groups is presented as boxplots in Figure S4. No significant results were found, neither for K_ep, TM_ nor other parameters. The box plots in Figure S5 show how std and skewness of K_ep, TM_ distribution changes with T stage. Again, no significant results were found, neither for K_ep, TM_ nor other parameters. A negative trend was found for std K_ep, TM_ with increasing T-stage (p-value $\geq$ 0.18). K_ep, TM_ was chosen to illustrate the results, as it was the only parameter that showed significant results in the T-stage analysis of the lesion mean, and strongest trend for association with T stage in the std analysis.

Other studies have found that std or skewness were potential prognostic biomarkers [2,3]. Therefore, it may still be interesting to investigate these further in terms of outcome.


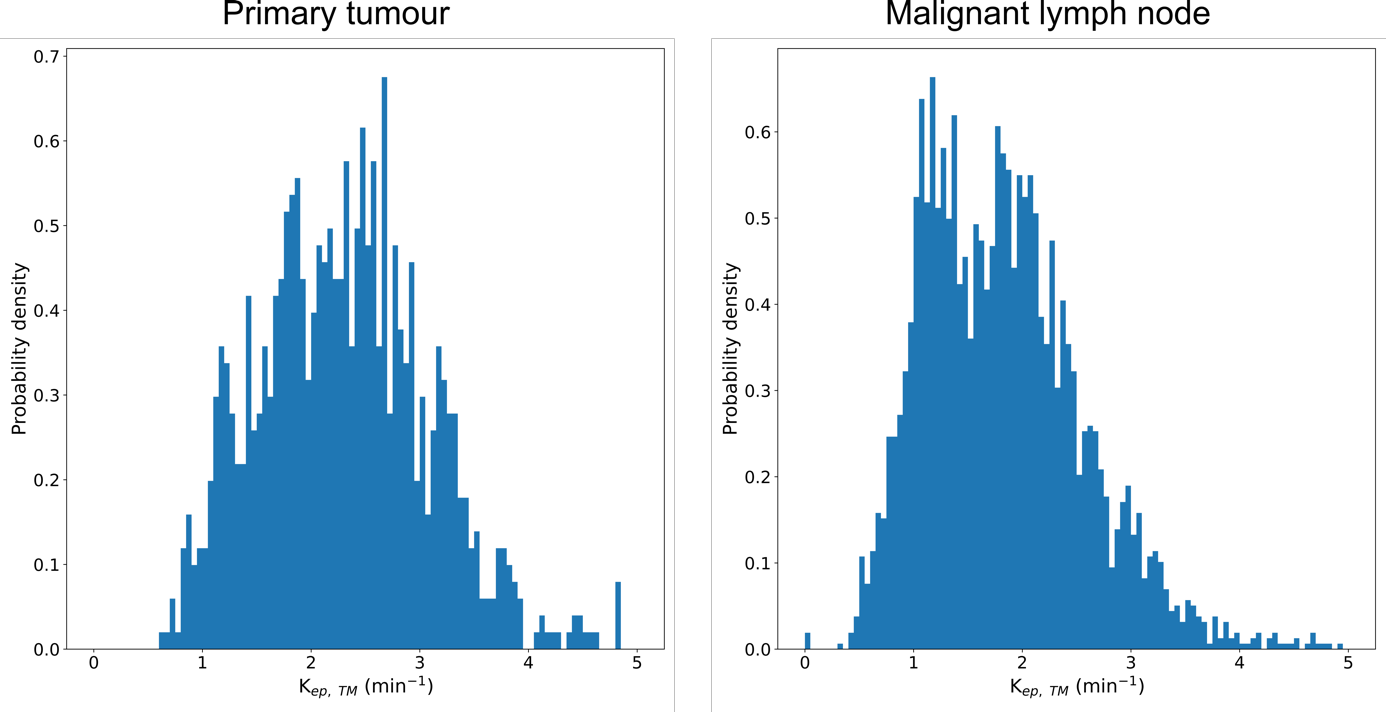


Supplementary Figure S3: Histograms of K_ep, TM_ for the primary tumour (left) and malignant lymph node (right) of one patient. K_ep, TM_ std and skewness were 0.77 and 0.33 in the primary tumor, and 0.73 and 0.70 in the lymph node.


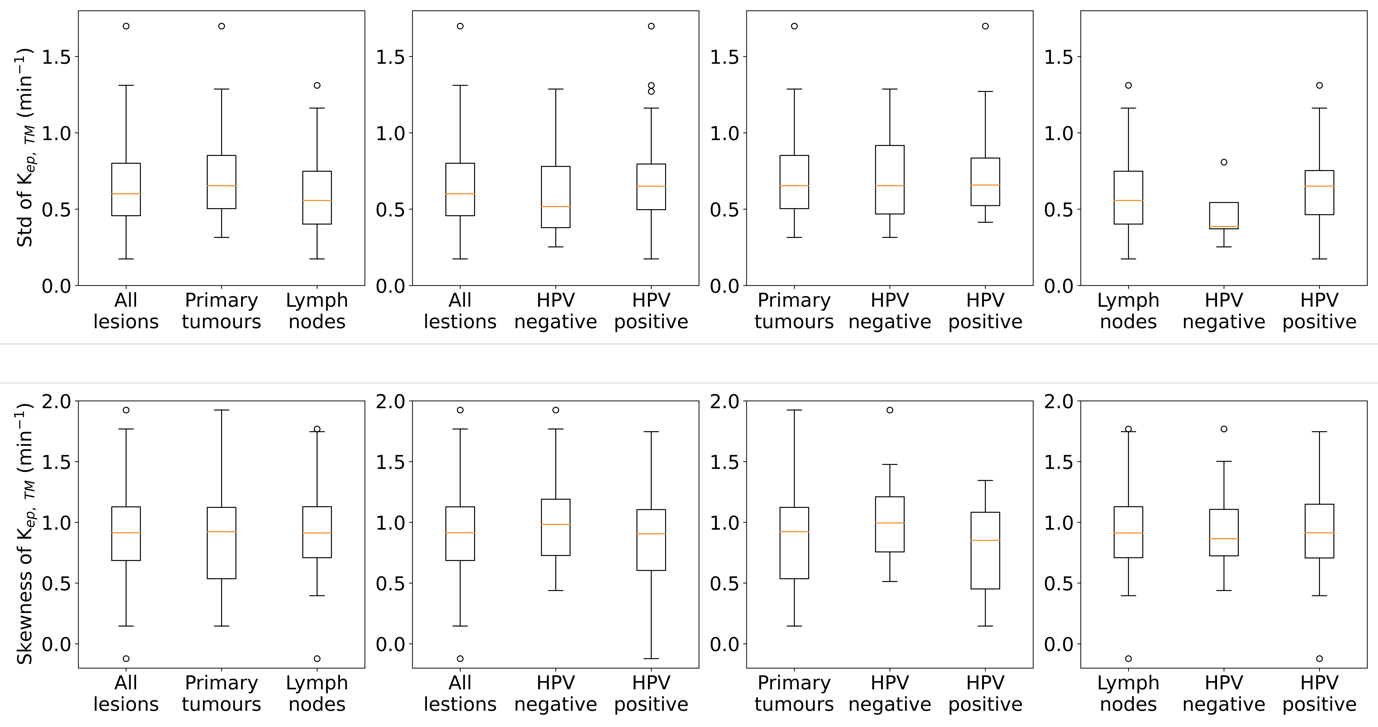


Supplementary Figure S4: K_ep, TM_ was calculated on a voxel-by-voxel basis for all lesions. The box plots show the standard deviation (std, top row) and skewness (bottom row) of the K_ep, TM_ distributions for primary tumours vs. malignant lymph nodes (first column), HPV negative vs. HPV positive lesions (second column), HPV negative vs. HPV positive primary tumours (third column) and HPV negative vs. HPV positive malignant lymph nodes (fourth column).


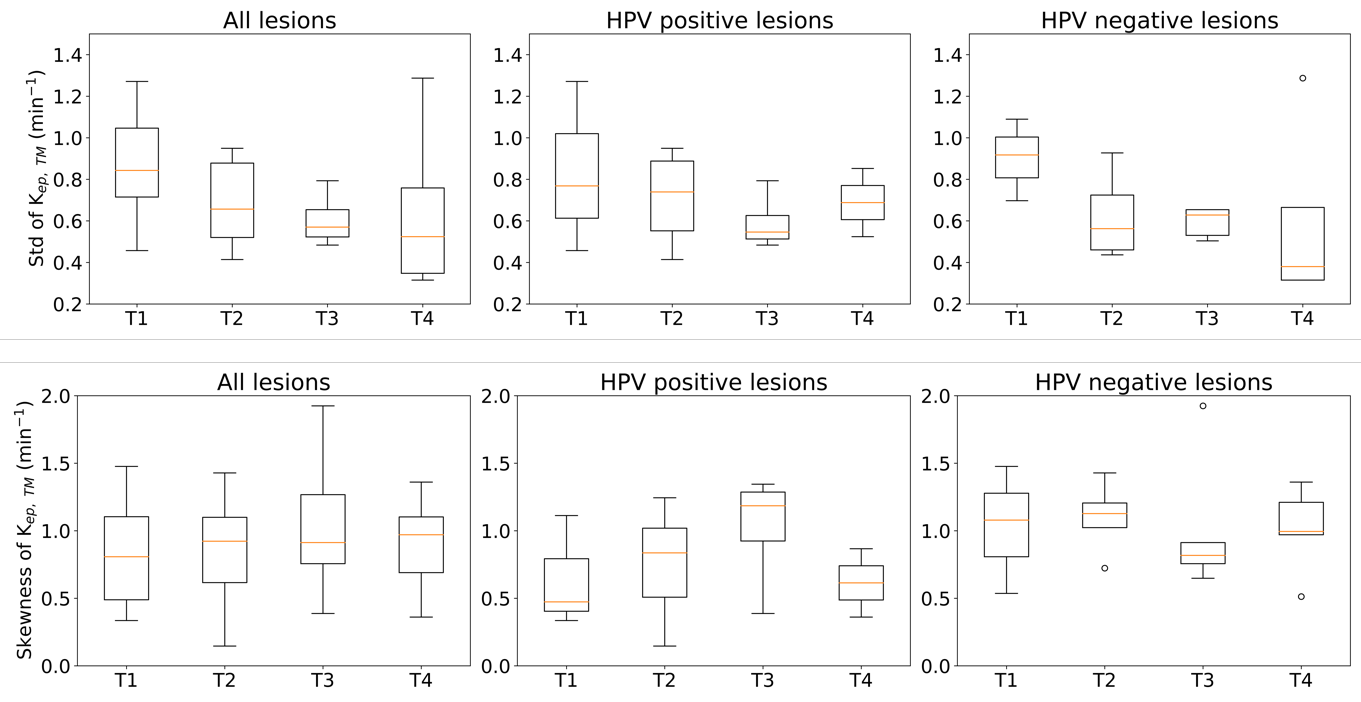


Supplementary Figure S5: K_ep, TM_ was calculated on a voxel-by-voxel basis for all lesions. The box plots show the difference in standard deviation (std, top row) and skewness (bottom row) of the K_ep, TM_ distributions between different T staged primary tumours where either all primary tumours (left), HPV positive (middle) or HPV negative (right) primary tumours were included.

# References

[1] Parker GJM, Roberts C, Macdonald A, Buonaccorsi GA, Cheung S, Buckley DL, et al. Experimentally-derived functional form for a population-averaged high-temporal-resolution arterial input function for dynamic contrast-enhanced MRI. Magn Reson Med 2006;56:993–1000. https://doi.org/10.1002/mrm.21066.

[2] Shukla-Dave A, Lee NY, Jansen JFA, Thaler HT, Stambuk HE, Fury MG, et al. Dynamic Contrast-Enhanced Magnetic Resonance Imaging as a Predictor of Outcome in Head and Neck Squamous Cell Carcinoma Patients with Nodal Metastases. Int J Radiat Oncol Biol Phys 2012;82:1837–44. https://doi.org/10.1016/j.ijrobp.2011.03.006.

[3] Tao X, Wang L, Hui Z, Liu L, Ye F, Song Y et al. DCE-MRI Perfusion and Permeability Parameters as predictors of tumor response to CCRT in Patients with locally advanced NSCLC. Sci Rep 2016;6:e35569. https://doi.org/10.1038/srep35569.
